# Our experience with oral and maxillofacial soft tissue injuries at Mbeya zonal referral hospital: A report of two cases and literature review

**DOI:** 10.1016/j.ijscr.2025.111731

**Published:** 2025-07-24

**Authors:** Msafiri Birigi, Bogias Mwamgunda, Harun Malaso, Mercy Bingileki, Jimmy Olomi

**Affiliations:** a Dental Department, Mbeya Zonal Referral Hospital, P.O. Box 419, Mbeya, Tanzania; b Mbeya College of Health and Allied Sciences, University of Dar es Salaam, P.O. Box 608, Mbeya, Tanzania; c Dental Department, Mirembe Mental National Hospital, P.O Box 910, Dodoma, Tanzania

**Keywords:** Oral and maxillofacial, Soft tissue injury, Wound debridement, Immediate management, Quality of life, Case report

## Abstract

**Introduction:**

Maxillofacial injuries pose significant challenges due to their impact on both function and aesthetics, with early and appropriate care the patients have good outcomes.

**Case presentation:**

We present two cases of severe facial trauma successfully managed at Mbeya Zonal Referral Hospital. The first case involved a patient with multiple lacerations and soft tissue injuries following a road traffic accident, which was managed with meticulous wound debridement and layered closure, leading to excellent healing. The second case was a 23-year-old male with a large avulsed facial wound sustained in a high-impact motorcycle collision. Timely surgical intervention, including thorough debridement, hemostasis, and structured wound closure, resulted in remarkable esthetic and functional recovery.

**Discussion:**

In the management of oral and maxillofacial trauma, both esthetic restoration and functional rehabilitation are critical goals. Although the optimal wound closure time is within 4–6 h post-injury, our case demonstrated excellent healing despite a 10-h delay. Effective management involved thorough wound decontamination, conservative debridement, and irrigation with antiseptic solutions to prevent infection. Surgical closure followed esthetic and functional principles, ensuring proper alignment of facial structures without complications such as parotid duct or facial nerve injury.

**Conclusion:**

Both cases highlight the importance of timely intervention, meticulous surgical technique, and comprehensive postoperative care in achieving favorable outcomes for maxillofacial trauma patients.

## Highlights

• Early and thorough wound management improves outcomes.
• Severe injuries with disfigurement management in resource limited settings.
• The role of multidisciplinary care in maxillofacial trauma.

## 1 Introduction

Oral and maxillofacial region is the physical identity for an individual, any catastrophic damage to this region can physically and psychologically traumatize the afflicted person [1,2].

Effective and definitive management, with thorough esthetic restoration and functional rehabilitation is of paramount important. Therefore, the emphasis in management of maxillofacial injury victims should be on an early, definitive, and aggressive surgical repair and reconstruction of the facial skeleton, thus restoring quality of life [1].

Due to the complexity of oral and maxillofacial region, it is essential to anticipate the injuries in various structures underneath the wound. The first chance is the best chance for repair as it decides the outcome. The surgeon needs to understand the mechanisms of wound healing and the art of soft-tissue repair [3].

The fundamental constituents of the management of oral and maxillofacial injuries include stabilization of the patient, complete examination of the wound, thorough wound irrigation and debridement of necrotic tissue, preservation of all viable tissue, tension-free closure, and realignment of important facial esthetic structures. Special consideration must be given to injuries of functional structures such as the facial nerve, ductal systems or organs, and ensuring proper management of these structures [[4], [5], [6], [7]].

By sharing these cases, we aim to emphasize key principles in trauma management, including early intervention, appropriate surgical techniques, and the importance of post-operative follow-up. These cases serve as valuable learning points for optimizing trauma care in resource-limited settings, where timely and effective management can significantly improve patient outcomes.

This cases report has been reported in line with the SCARE checklist [8].

## 2 Case 1

### 2.1 History

**Fig. 1:**
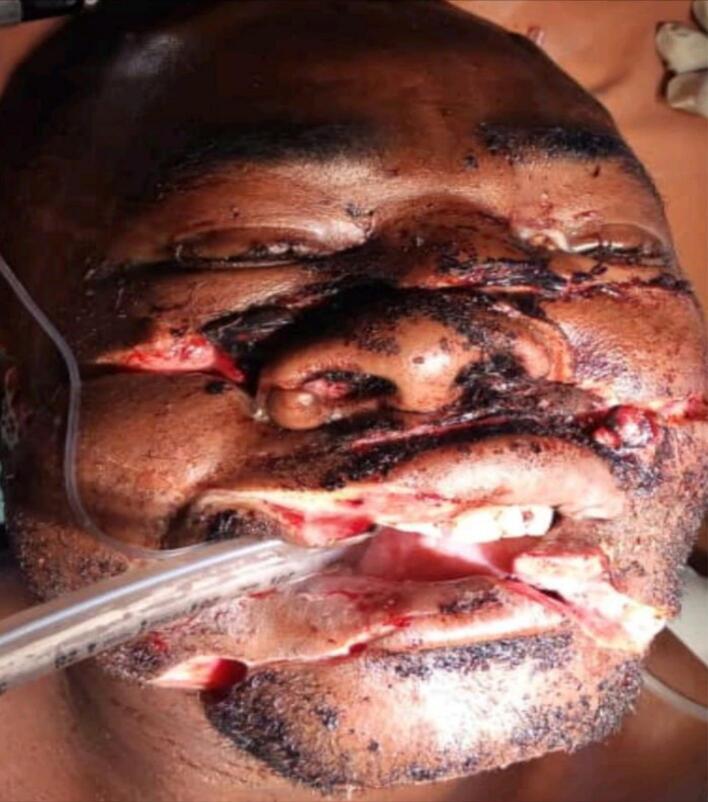
Showing the disfigured face of case one before surgery.

A 35-year-old peasant was received at our emergency department as a referral case from a district hospital. The patient had multiple large lacerated wounds on the face (Fig. 1).

The patient reported to be assaulted by a gang of people who used sharp objects and sustained injury to his face. During the incidence he felt down on his face and lost consciousness for unknown period. There was bleeding from the wounds mouth and nose, he was taken to the district hospital where the bleeding was controlled by sutures and pressure packs with a gauze then plain skull radiograph and full blood picture (FBP) were done before he was referred to us.

At our emergency department, the patient was confused with GCS of 14/15, Blood pressure = 140/78 mmHg, Temperature = 37.0 degree of Celsius, S*PO*_*2*_ = 96 %, Respiratory rate = 14 breath /min, Pulse rate = 52 beat/min but he was slightly pale and the pack of dressing gauze was soaked with blood. Quick history and examination were done and the patient had no any comorbidities.

### 2.2 Local examination

There was facial disfigurement due to Multiple large wounds on the face as seen in Fig. 1. There were three cut wounds, the upper one extending from the right to left malar regions with approximate length of 10 cm and depth of 1.5 cm, involving the soft tissues, the nasal bones and zygomatic bones fracture both sides. The middle wound was extending from the right cheek through the upper lip to the left cheek with approximate length of 9 cm and depth of 1 cm, involved the soft tissue only. The third cut wound was extending from the right cheek through the lower lip and symphysial part of the mandible to the left cheek with approximate length of 10 cm and depth of 1.5 cm involving the soft tissues and simple, non-displaced fracture of the symphysial part of the mandible. The facial artery and vein on the right side were destroyed. Otherwise, the other maxillofacial bones were intact without any sign of fracture. The temporomandibular joints were normal.

### 2.3 Investigations

The CT-Scan was performed to rule out any brain injury and showed that there was fracture of zygomaticomaxillary complex fracture (ZMC) both sides, nasal bones and body of the mandible (images unavailable). Full blood picture was essentially normal except the Hb was low (8.7 g/dl), blood grouping, cross matching (blood group B+) and electrolytes was within normal range (BUN = 9.85 mmol/l, Creatinine = 86micromol/l, Na+ = 140.95 mmol/l and K+ **=** 4.03 mmol/l).

The patient was given intravenous Paracetamol 1 g stat for his pain, Metronidazole 500 mg stat and Ceftriaxone 1 g stat as antibiotic prophylaxis. The preparation of surgical toilet and suture under general anesthesia was immediately done and the patient was sent to the theatre within 1 h.

### 2.4 Intraoperative management

The operation was done 10 h post injury by a team of oral & maxillofacial and ENT surgeons. Under GA the wound debridement was done by removing all foreign bodies and cutting all dead tissues to expose the living parts of tissues. The wound was irrigated with copious amount of normal saline followed by povidone iodine before closure of the wound. Open reduction and fixation of the fractured fragments of the facial bones was done using titanium mini plates. The reconstruction of soft tissues on the face started by apposing the tissues by layers, starting suturing the periosteum and muscles using vicryl 2/0 while the mucosa was sutured using vicryl 3/0. The skin was closed by simple interrupted sutures using 3/0 polypropylene with special care in approximating the vermilion borders of the lips. The skin around the nose was repaired by placing key sutures at the rim of the nose, using 6/0 polyethylene, before the residue of the closure. A nasal stent was inserted in both nostrils with external splinting. Transfusion of two units of blood was done, one unit intraoperative and another unit post-operative. Intraoperative medications were Iv Metronidazole 500 mg, Iv Ceftriaxone 1 g and Iv Dexamethasone 8 mg stat, Normal saline 1 l, Ringer lactate 1 l and Dextrose 5 % -1 l. The procedure took about 5 h.

Post -operative medications were: Iv Metronidazole 500 mg 8-hourly for 2 days, Iv Dexamethasone 8 mg 8-hourly for 2 days, Iv Ceftriaxone 1 g 12-hourly for 2 days, Iv Diclofenac 75 mg 8-hourly for 2 days and Iv dextrose 5 % 1 l- 12hourly for 24 h. The patient was discharged on the fourth day postoperative in a stable condition and was able to eat per oral. The patient was given oral antibiotics (Metronidazole and Amoxycillin and Clavulanic acid), analgesics (Ibuprofen), anti-inflammatory (Dexamethasone) and Hydrogen peroxide mouth wash for more five days. The patient was given an appointment to return to the hospital for stich removal after 10 days.

**Fig. 2:**
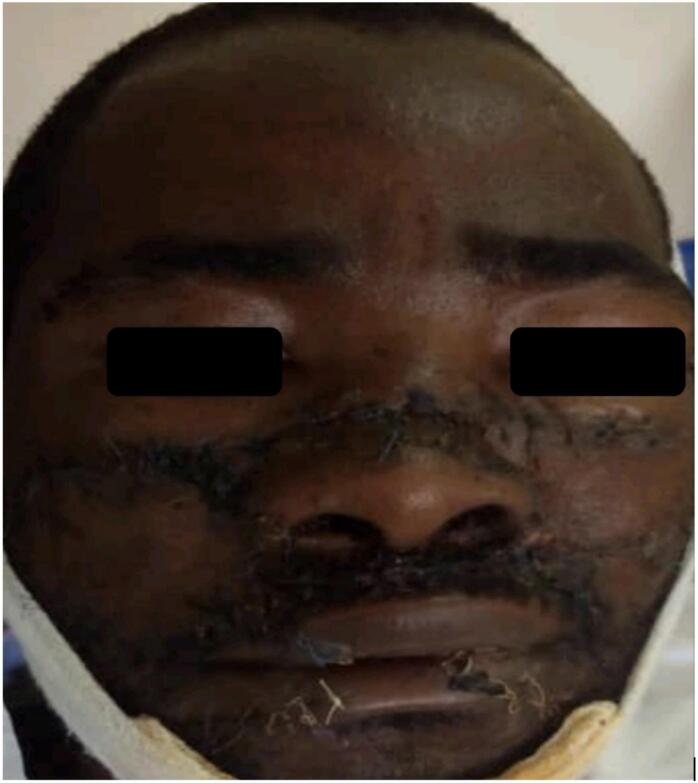
Showing 10 days post-operative with healing wounds stitches and nasal stents removed.

After 10 days the patient returned to the hospital for stitch and nasal stent removal. The stitch was removed and the healing was progressing well and the wound was suitably closed Fig. 2.

**Fig. 3:**
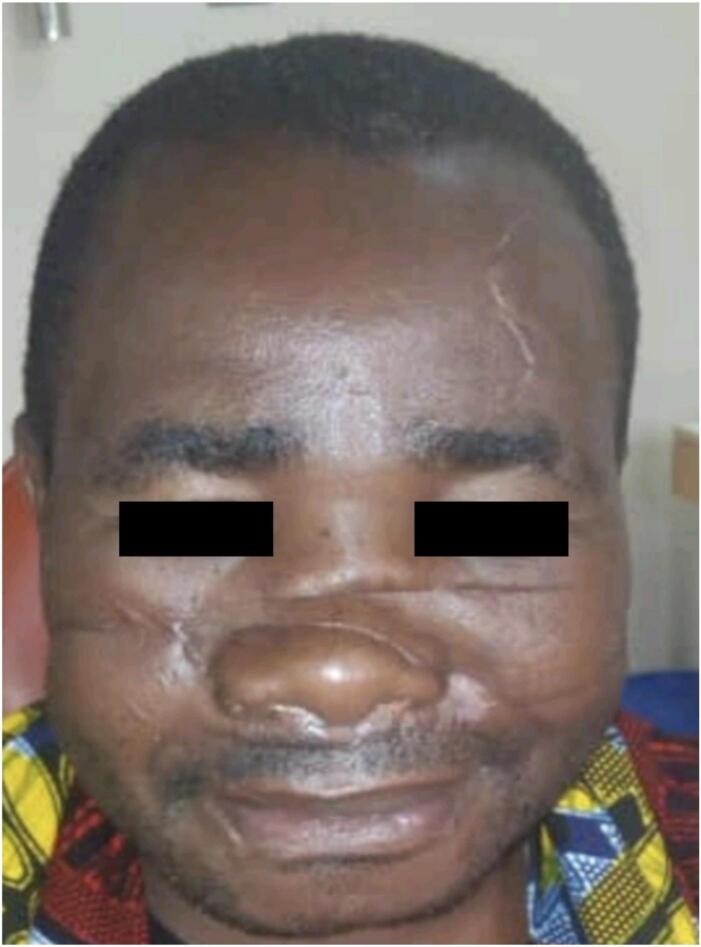
Patients face with well healed scars.

He was given an appointment to come back for view after 21 days, where we found the healing progress was superb with an acceptable scar size considering the injury as depicted in Fig. 3. The patient was then transferred to the ENT department for possible rhinoplasty surgery.

## 3 Case 2

### 3.1 History

A 23-year-old male patient was received at our emergency department as a referral case from a regional referral hospital with an injury to his face. He was a motor cyclist at a high speed, when he collided with a fast-moving semi-trailer lorry. After the collision he fell down on the tarmac road to his right side of the face, he lost consciousness for sometimes, bleeding from the wound mouth and nose but did not bleed from his ears no history of vomiting. He was taken to the Regional Referral Hospital (NRRH) where bleeding was controlled by pressure pack with a gauze and one unit of blood was transfused then plain skull radiograph, FBP, Blood grouping and cross matching were done.

After stabilizing the patient, he was referred to our facility (MZRH) for further investigations and possible management. Upon arrival patient was stable with GCS score of 14/15, vital signs of Blood pressure = 143/62 mmHg, Temperature = 37.0 degree of Celsius, S*PO*_*2*_ = 96%, Respiratory rate = 14 breath /min, Pulse rate = 52 beat/min but he was pale and the pack of dressing gauze was soaked with blood. Quick history and examination were done and the patient had no any comorbidities.

### 3.2 Local examination

**Fig. 4:**
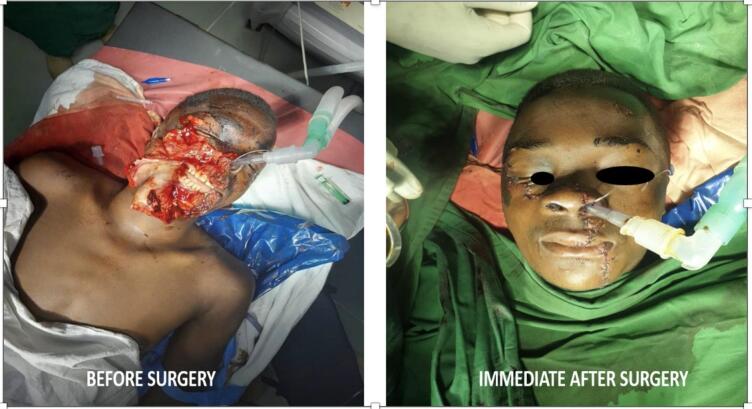
The left image is a preoperative view of an avulsed laceration on the right side of the patient's face, while the right image displays the immediate postoperative look.

There was facial disfigurement, due to a large lacerated and avulsed wound on right side of the face Fig. 4. The lacerated wound was extending from the lower border of the body of the mandible on the left side cutting through the lower lip, upper lip, right nostril involving the cartilage, right cheek, the right malar region to the inferior rim of the right eye, with approximate length of 17 cm, superior-inferiorly with a depth of 1 cm. The maxilla and mandible were exposed from the midline to the level of third molar on the maxilla right side to the level of first molar on the left side of the mandible, exposing both upper and lower teeth of that region. The facial vein and artery on the right side were destroyed. There was a small free bone fragment from a buccal plate of the mandible attached to the soft tissue. Otherwise, the maxillofacial bones were intact without any sign of fracture. The temporomandibular joints were normal.

### 3.3 Investigations

The CT-Scan was performed to rule out any brain injury and bone fractures but the results were normal (images are unavailable). Blood sample for Full Blood Picture was essentially normal except the Hemoglobin level was low (7.7 g/dl), blood grouping, cross matching (A+) and electrolytes was within normal range (BUN = 9.85 mmol/l, Creatinine = 86micromol/l, Na+ = 140.95 mmol/l and K+ **=** 4.03 mmol/l).

The patient was given intravenous Paracetamol 1 g stat for pain relief, Metronidazole 500 mg stat and Ceftriaxone 1 g stat as antibiotic prophylaxis. The preparation of surgical toilet and suture under general anesthesia was immediately done and the patient was sent to the theatre within 1 h.

### 3.4 Intraoperative management

The operation was done 10 h post injury. Under GA the wound debridement was done by removing all foreign bodies including a bone fragment of the buccal plate and cutting all dead tissues to expose the living parts of tissues. The wound was irrigated with large amount of normal saline followed by povidone iodine before closure of the wound. The reconstruction of the face started by apposing the layers starting suturing the periosteum and muscles using vicryl 2/0 while the mucosa was sutured using vicryl 3/0. The skin was closed by simple interrupted sutures using Size 3/0 polypropylene with special care in approximating the vermilion borders of the lips. The skin around the nose was repaired by placing key sutures at the rim of the nose, using 6/0 polyethylene, before the residue of the closure. A nasal stent was inserted in the right nostril. Transfusion of two units of blood was done, one unit intraoperative and another unit post-operative. Intraoperative medications were Iv Ampiclox 500 mg and Iv Dexamethasone 8 mg stat, Normal saline 1 l, Ringer lactate 1 l and Dextrose 5 % -1 l. The procedure took about 4 h to be completed.

### 3.5 Post-operative management

The patient was kept on Iv Metronidazole 500 mg 8-hourly for 2 days, Iv Dexamethasone 8 mg 8-hourly for 2 days, Iv Ampiclox 500 mg 8-hourly for 2 days, Iv Diclofenac 75 mg 8-hourly for 2 days and Iv dextrose 5 % 1 L- 12hourly for 24 h. The patient was discharged on the third day postoperative in a stable condition and was able to eat per oral. The patient was given oral antibiotics (Metronidazole and Amoxycillin and Clavulanic acid), analgesics (Ibuprofen), anti-inflammatory (Dexamethasone) and Hydrogen peroxide mouth wash for more five days. (We routinely prescribe ceftriaxone but this time it was not available so instead we used amoxycillin and clavulanic acid) The patient was given an appointment to return to the hospital for stich removal after 10 days.

**Fig. 5:**
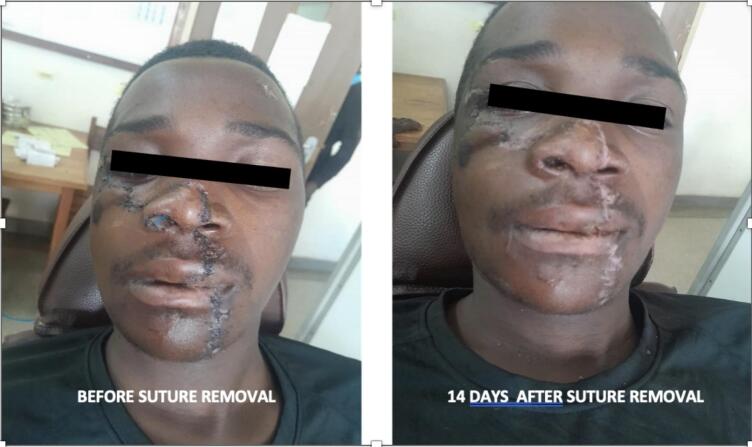
The left image shows the healing progress 10 days postoperatively, while the right image depicts the outcome 14 days after suture and stent removal (24 days postoperatively).

After 10 days the patient returned to the hospital for stitch and nasal stent removal. The stitch was removed and the healing was progressing well and the wound was suitably closed see Fig. 5. He was given an appointment to come back for review after 14 days, where we found the healing progress was superb Fig. 5.

## 4 Discussion

The major goals for the effective and absolute management of oral and maxillofacial trauma are esthetic restoration and functional rehabilitation. Our patients apart from losing hope, esthetic and function of some of orofacial organs such as nose and lips, the prompt management which was done ensured restoration of esthetic and functions. One of the key components of the management of the orofacial injury is early surgical toilet and suturing, of which the time suggested was 4–6 h post injury for better results [3,9], in our case the time for the wound management was 10 h with brilliant outcomes. Early surgical management requires thorough decontamination and debridement with the removal of all foreign debris before the wound is closed. Debridement should still be conservative, and any questionably viable tissue should be left alone. Thorough wound debridement with adequate irrigation with normal saline mixed with antibiotics or antiseptics like chlorohexidine ensures cleanliness of the wound and ensures prevention of postoperative infection. [[9], [10], [11]]. Intraoperatively we did cleanliness of the wound through irrigation of large volume of saline, conservative trimming of nonviable tissue margins and tissues, and painting of povidone iodine to the wound before closure of the wound. This has been providing with better healing without postoperative infection as observed also in the current case.

The closure of the wound needs to follow the esthetic and functional principals. When repairing the skin of the nose, sutures should be placed to align the nasal landmarks to ensure an appropriate shape and contour of the nose [12]. In case of cheek lacerations without significant soft tissue loss should be repaired primarily with a layered closure to approximate the mucosa, muscle, and skin. Repair of full thickness lip lacerations requires the careful approximation of all anatomic structures in order to maintain oral competence and optimizing esthetic outcome [[13], [14], [15], [16]]. In the present case there was no injury to the parotid gland duct and obvious facial nerves injury. We followed all the principles of repair of the soft tissue injury which provided early healing with restoration of esthetic, functions and improving quality of life to the patient.

## 5 Conclusion

Oral and maxillofacial injuries usually lead to esthetic, functional and psychological catastrophe. Early management of the patient with thorough wound debridement and good art of wound closure ensures early healing of the wound with good esthetic and functional restoration.

## Author contribution

Dr. Msafiri Birigi, Dr. Bogias Mwamgunda and Dr. Harun Malaso performed the procedures. Dr. Msafiri Birigi, Dr. Mercy Bingileki and Dr. Jimmy Olomi were involved in the writing of this case report.

Dr. Jimmy Olomi did all the correspondence.

## Consent

Written informed consent was obtained from the patient to publish this case report and accompanying images. A copy of the written consent is available for review by the Editor-in-Chief of this journal on request.

## Ethical approval

Our hospital (Mbeya zonal referral hospital) does not require ethical approval for case report.

## Guarantor

Dr. Msafiri Birigi.

## Research registration number

Not applicable.

## Funding

None.

## Conflict of interest statement

The authors declare that they have no known competing financial interests or personal relationships that could have appeared to influence the work reported in this paper.
